# Supplementary material for: Epidemic Diffusion Network of Spain: A Mobility Model to Characterize the Transmission Routes of Disease
Source: Int J Environ Res Public Health. 2023 Feb 28;20(5):4356. doi: 10.3390/ijerph20054356 (PMC10001675; doi:10.3390/ijerph20054356)
Supplement: Supplementary file 1 [file ijerph-20-04356-s001.zip › ijerph-2206945-supplementary.pdf]

Supplementary Materials

Epidemic Diffusion Network of Spain: A Mobility Model to Characterize the Transmission Routes of Disease

Javier Del-Águila-Mejía [1,2,3,*], David García-García [2,4], Ayelén Rojas-Benedicto [2,4,5], Nicolás Rosillo [6], María Guerrero-Vadillo [2,4], Marina Peñuelas [2,4], Rebeca Ramis [2,4], Diana Gómez-Barroso [2,4] and Juan de Mata Donado-Campos [1,4]

[1] Departamento de Medicina Preventiva y Salud Pública y Microbiología, Facultad de Medicina, Universidad Autónoma de Madrid. C. Arzobispo Morcillo 4. 28029 Madrid, Spain
[2] Centro Nacional de Epidemiología, Instituto de Salud Carlos IIII, Calle de Melchor Fernández Almagro 5, 28029 Madrid, Spain
[3] Servicio de Medicina Preventiva, Hospital Universitario de Móstoles, Calle Río Júcar s/n, 28935 Móstoles, Spain
[4] Consorcio de Investigación Biomédica en Red de Epidemiología y Salud Pública (CIBERESP), Calle Monforte de Lemos 3-5, 28029 Madrid, Spain
[5] Universidad Nacional de Educación a Distancia (UNED), Calle de Bravo Murillo 38, 28015 Madrid, Spain
[6] Servicio de Medicina Preventiva, Hospital Universitario 12 de Octubre, Avenida de Córdoba s/n, 28041 Madrid, Spain
* Correspondence: javidelaguila@gmail.com

Content table

Defining a Mobility Matrix to sum up mobility in Spain 2
Stable matrix of connections 6
From the Mobility Matrix to the Epidemic Diffusion Network 7
Table S2. Epidemic Diffusion Network measures by province and community 9
Community structure from the EDN: hierarchical clustering 10

Defining a Mobility Matrix to sum up mobility in Spain

To define a Mobility Matrix (MM) implies the assumption that stable patterns can be derived from brute daily number of travels in Spain. Figure 1 in main text depicts this aggregated brute mobility, but it fails to reflect the behavior at the underlying province level which we considered the geographical unit of study.

Between any two provinces m and n, there is a flux of total travelers F_{nm} moving from m to n, and F_{mn} in the opposite direction. These fluxes allow to understand true mobility dynamics and their characteristics. We will be using data from Madrid (capital of Spain) and one adjacent province, Guadalajara, to discuss some properties relevant to posterior analysis. It is important to notice that Madrid is a mega-region of 6,6 million inhabitants with a daily mean of around 450.000 travelers (14,6% of its total population), while Guadalajara is only 260.000 inhabitants with around 100.000 daily travelers (39% of its population).

Figure S1 depicts weekly travelers between the two provinces during 2020 and daily travelers during the summer. Both fluxes show an almost perfect overlap between the two provinces. We can observe several effects: Weekly-aggregated travels follow the same national trend shown in figure 1 in the main text, affected by travel restrictions put in place by the government. When observing the daily mobility pattern, first we notice the weekend pattern with peak mobility over Fridays and Sundays, and also a flux being of higher magnitude (the one from Madrid). Finally, a seasonal variation can be seen during the month of August, with a decline in regular mobility that is restored in September.

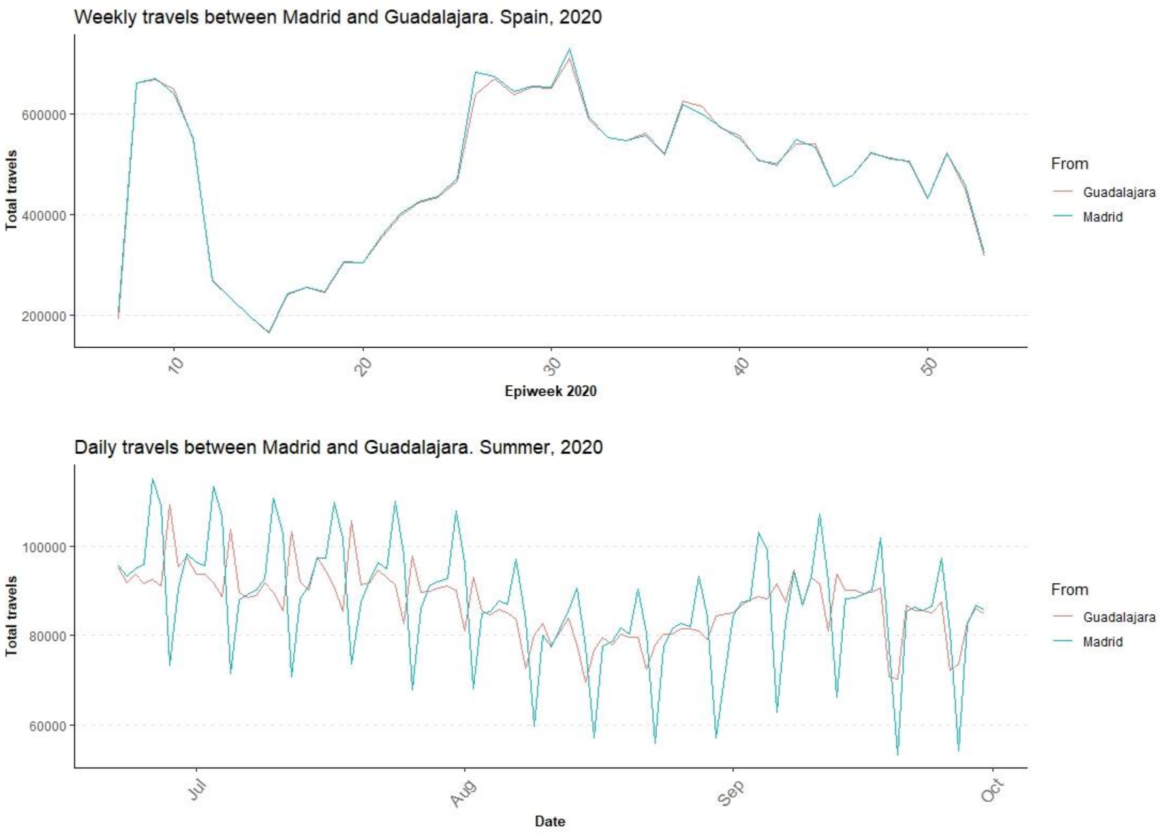

Figure S1. Travels between Madrid and Guadalajara during 2020

So, we can describe multiple sources of mobility variance: national or higher order (travel restrictions in place), seasonal (summer time) and weekday-related variation (weekends). For the two provinces, sharing these fluxes of travelers must have different implications given their enormous differences. To further understand them, we convert the fluxes F_{nm} to the percentage those travelers represent among all people leaving the provinces, P_{nm}:

$$\boldsymbol{P_{nm}} = \frac{Total\ travelers\ moving\ from\ m\ to\ n}{All\ people\ leaving\ m\ to\ any\ other\ province}.$$

Thus, P_{nm} represents the weight that a given flux of travelers has for a province, a measure of the relative importance of the connection between a pair of provinces in terms of mobility. When turned into the percentages P_{nm}, overlapping fluxes F_{nm} between Madrid and Guadalajara now acquire a different magnitude and a new significance (fig S2)

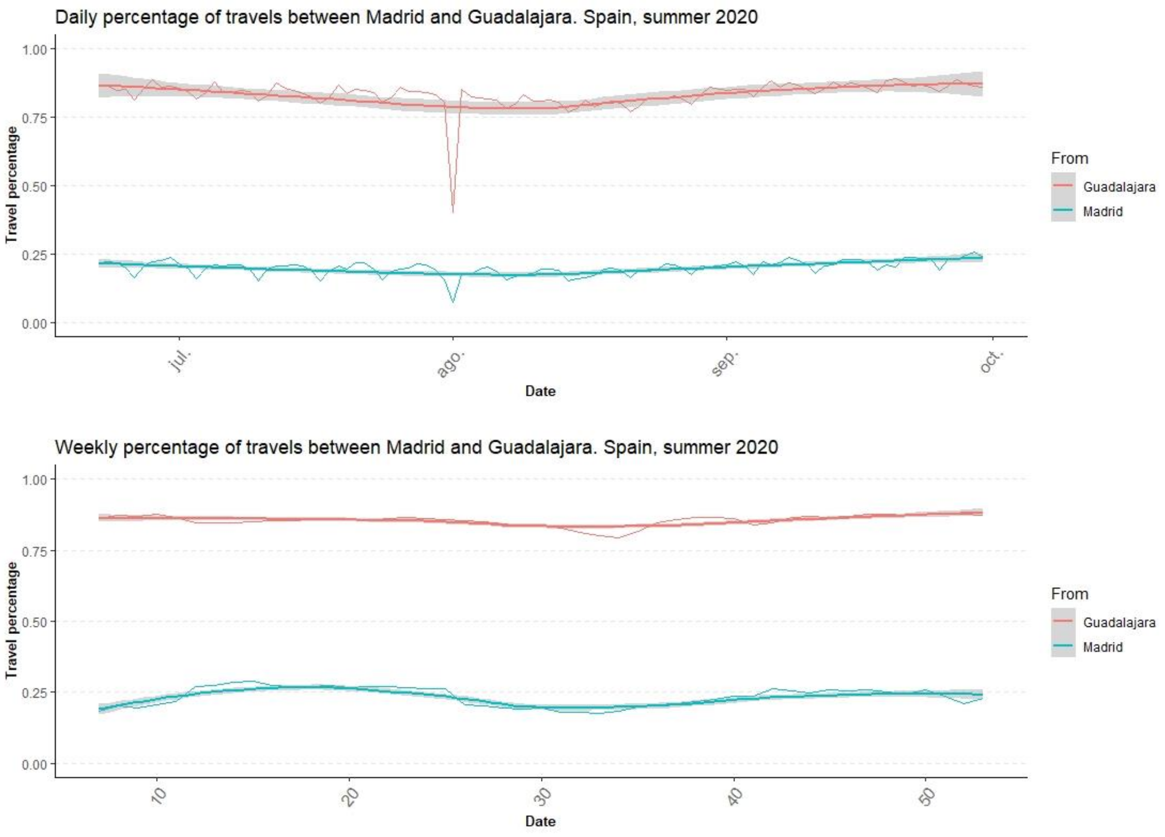

Figure S2. Percentage of daily/weekly travelers between Madrid and Guadalajara

Initially overlapping fluxes now represent a very different share of each province's travels. While travels from Guadalajara represent almost an 80% of their total travels, for Madrid this is only a bare 23%. This shows how very similar fluxes F do not translate to similar percentages of fluxes P, so:

$$F_{nm} = F_{mn}$$

but,

$$P_{nm} \neq P_{mn}.$$

This has strong implications in transmission phenomena: symmetrical fluxes (individual travelers) now imply asymmetrical weights and unequal transmission probabilities. The "cost" of traveling from one province to the other differs and a given spreading event has much higher chances to move from the small province of Guadalajara to Madrid than vice-versa. So, for every possible emanating province, the set of its connections expressed as the percentages P represents the first approximation to our MM. If we take a look to Madrid's and Guadalajara´s (fig S3) distributions of all P emanating from each one:

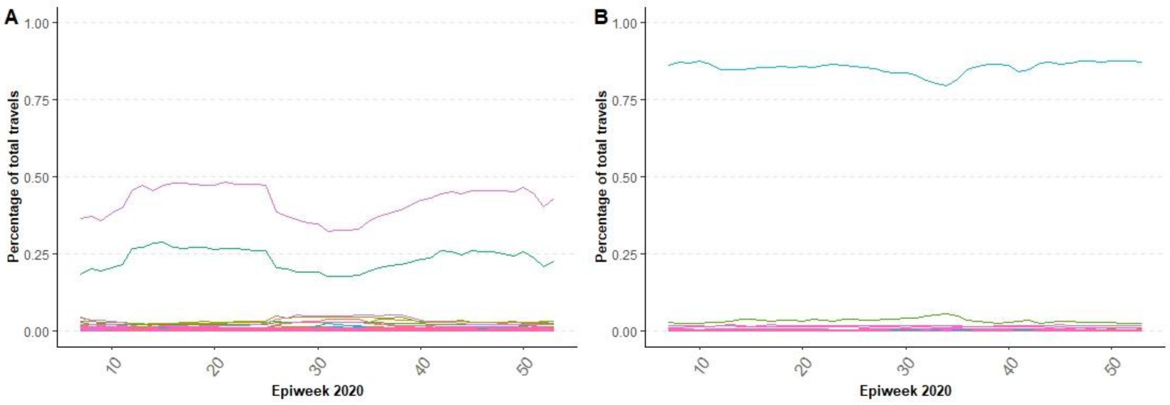

Figure S3. Weekly percentage of travels from Madrid (A) and Guadalajara (B) by epiweek, 2020

Guadalajara sends most of its travelers (83%) to only one place, Madrid. Madrid only sends back its 23%, and another 45% to a different neighboring province, Toledo. One and two main connections, respectively. The rest of connections from both provinces represent a multiplicity of very low-value connections that, in practice, have little to no effect on any hypothetical transmission phenomena.

From these properties we can classify each province according to the distribution of all their outgoing connections and, also, all pairs of provinces by their relationship. Figure S4 shows other examples of pair-to-pair connections of different magnitude and figure S5, the set of all connections for other provinces. Percentages P_{nm} remain more stable through time than the raw fluxes F_{nm}, and therefore allow to uncover inherent characteristics associated to mobility patterns in Spain.

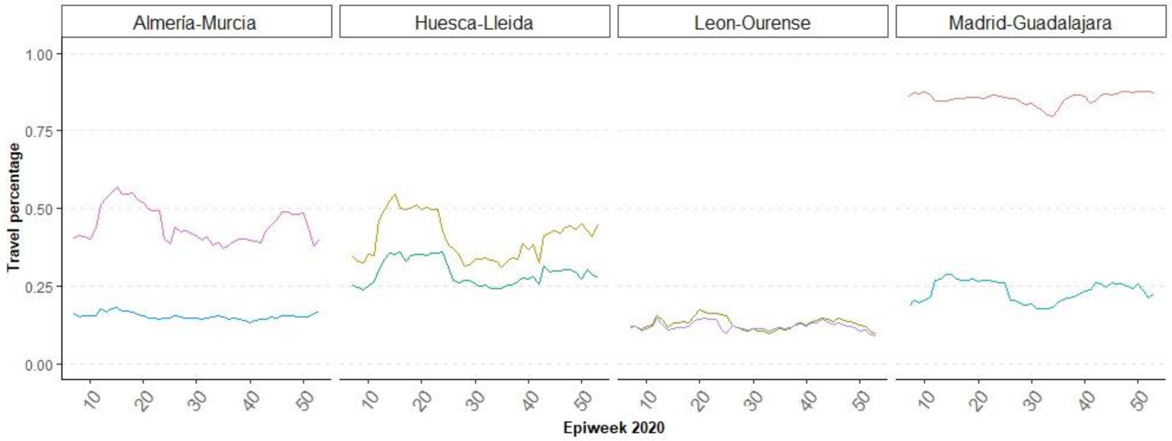

Figure S4. Daily percentage of travels between two provinces. Spain, 2020

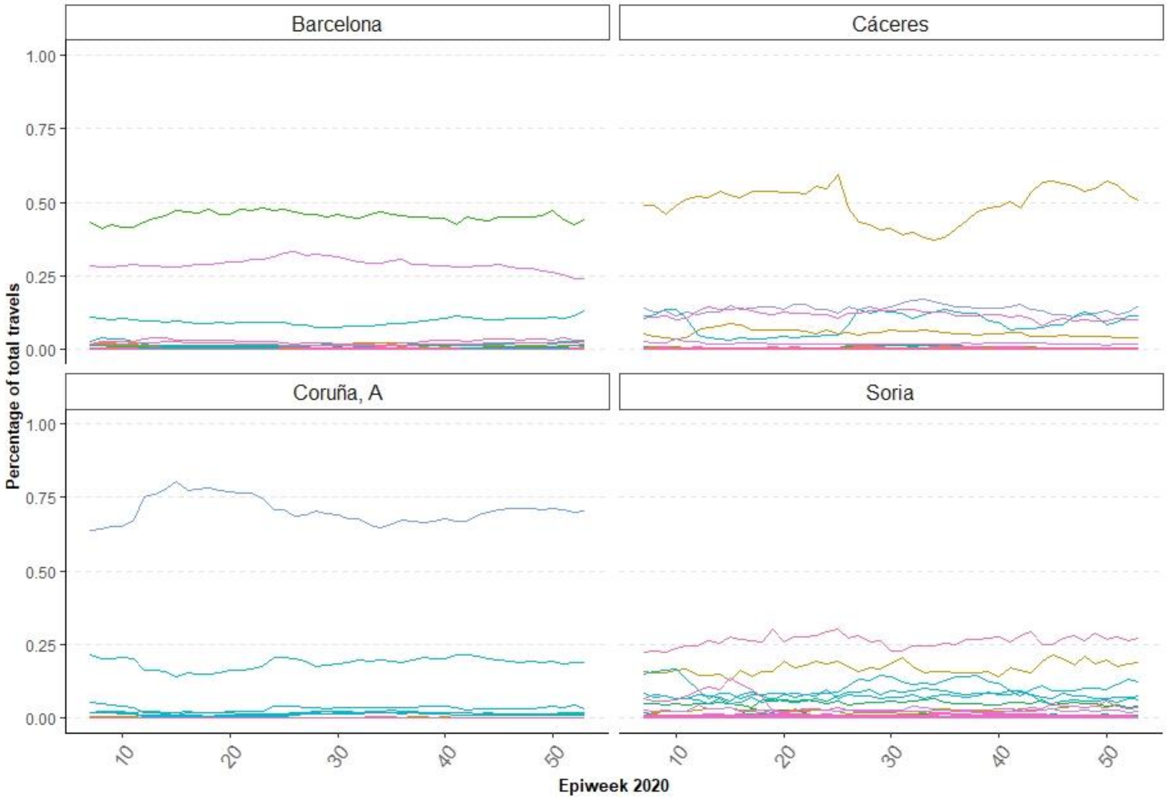

Figure S5. Weekly percentage of travels from each province

Each province has a unique set of relations with their neighbors. If we want to study transmission phenomena, this is of crucial importance since diffusion usually takes place forward. We can look at the distribution of all percentage-connections in the network to find a mean value of 3% and a median of 0.2%. That means that, even if people move through all provinces, it is clear that there are a set of important fluxes that will determine geographical mobility patters and thus, govern transmission phenomena linked to human mobility.

Stable matrix of connections

We have shown how percentages P_{nm} exhibit a smaller variation during a year period when compared with fluxes F_{nm} quantitatively (coefficient of variation 3.44 vs 4.98) and qualitatively (Figures S1-5). A mean P_{nm} matrix was calculated for the period 2020 as the mean percentage for every connection present at any time in the network. Secondary mean matrices were obtained for the following periods: February (control data previous to pandemic arrival), April (full lock-down mandated), Summer time (21st June - 30th August, relative normality) and November (second travel ban issued).

From each P_{nm} matrix above, Effective Distances (ED) were calculated as expressed in the main paper. To compare the four periods with the mean 2020 matrix, the ratio between ED for each secondary matrix and the mean matrix was obtained to test for divergences in values for each pair n, m. If the values compared were to be akin, their ratio

should be 1 or very close to 1. We explored mean and standard deviation for all compared periods with all of them being around 1 with small standard deviations, concluding that no relevant differences could be appreciated. April was the most differentiated period, as travelling was strictly prohibited. Thus, any P_{nm} matrix could be representative of the mobility patterns in Spain for the year 2020.

Table S1. Comparison of Effective Distance Matrixes during 2020.

	Mean ED for 2020			
	ED February	*ED April*	*ED Summer*	*ED November*
Ratio ED	1.01	0.98	1	1.01
Standard deviation	0.05	0.08	0.05	0.04

Therefore, we select the mean 2020 P_{nm} (and its ED derivative) as the Mobility Matrix that sums up qualitative properties, behavior and emerging properties of mobility patterns in Spain, regardless of time period, restrictions in place or other elements of variance.

From the Mobility Matrix to the Epidemic Diffusion Network

The defined MM is very rich in connections. Between the 52 Spanish provinces, 2264 connections arise. We saw how the mean (3%) and median (0,3%) P_{nm} values (for d_{nm}, these values are 7.17 and 7.54, respectively) of the MM pointed towards an absolute dominance of major travel fluxes through only a limited set of connections. If we focus on relevant connections only, e.g., those with P over 10% (or d_{nm} < 3.10), only 137 out of 2264 (6%) present connections arise. Therefore, to define a network model for studying geographical diffusion, only provinces connected by percentage of at least 10% of total travelers were considered. The connection can exist only in one direction or its reciprocal and the effective distance acts as the connection weight, pondering the relationships. This defines our Epidemic Diffusion Network (EDN) which we explore by means of network measures and communities, as explained in the main paper.

Table S2. Studied network measures and their epidemiological implications.

Metric	Type	Meaning	Epidemiology
**Degree**	Centrality	Number of connections each node has in the network. Can be divided into incoming and outgoing degree in directed networks	***Infection risk***. Nodes with higher degree/strength score tend to be at higher risk of exposure to an infection and, at the same time, will contribute more to the onward propagation in infection to a higher number of individuals. Super-spreader would represent the highest-degree individuals
**Strength**	Centrality	The weighted degree i.e., the sum of all connections of a node multiplied by the weight of that connection. Only for weighted networks.	
**Betweenness**	Global	Measure of the ability of an individual to connect the different components of a network. Measures how many times a node lies in the shortest path between any other two nodes of the network. Higher scoring nodes act as bridge between multiple network components	***Spread-capacitators***. Nodes with higher betweenness will have a relevant role in regulating the spread of an endemic disease within a population in network with higher substructure. They identify individuals that contribute the most to the diffusion of the disease to other communities or components of the network. In a social network, individuals who relate to different groups of otherwise isolated people would represent the better example.
**Communities**	Structural	A community within a network -or a subcommunity- is a cluster of nodes where interaction if more frequent between them compared with nodes outside the detected community. Many algorithms and methods exist for detecting communities, depending on the context of the study	***Clustering***. The epidemiological concept of grouping, or clustering, applies here. Some clusters are closer than others, but the higher the clustering degree, the more likely it is for a disease to spread slower through a network or to become trapped in one of the communities. It also identifies groups of individuals which may present some differential characteristic of interest or to have into consideration
**Modularity**	Structural	Global measure of how many of the network flow happens within detected communities rather than between them or, in other words, of the strength of the subdivision of a network in modules	Modularity influences the meaning of the other measures. In the presence of a high modularity network, an increased variance in centrality measures is expected since each community will conform its own relevant nodes. There is controversy regarding the relationship between modularity and the property of structural trapping of the network (the capacity to contain the disease within a community)

Adapted from Silk et al. (ref [25] in main text) with information summarized for the same source

Table S3. Epidemic Diffusion Network measures by province and community.

Province	Degree	Strength	Flow betweenness	Community
Madrid	11	25,6	65	Central-East community
Valladolid	9	24,2	259	Central-West community
Araba/Álava	8	20,2	140	North community
Málaga	8	19,3	64	South community
Sevilla	8	18	1231	South community
Valencia/València	8	19,3	840	Central-East community
Albacete	7	19,2	52	Central-East community
Ávila	7	19,9	76	Central-West community
León	7	20,1	553	Central-West community
Salamanca	7	19,3	1155	Central-West community
Bizkaia	7	15,6	385	North community
Zaragoza	7	17	797	Northeast community
Barcelona	6	10,6	90	Northeast community
Córdoba	6	15	50	South community
Granada	6	14,2	1057	South community
Gipuzkoa	6	15,5	37	North community
Lugo	6	14,1	166	Northwest community
Navarra	6	14	810	North community
Ourense	6	15,9	166	Northwest community
Asturias	6	15,6	527	Central-West community
Toledo	6	14,3	11	Central-East community
Zamora	6	15,4	148	Central-West community
Alicante/Alacant	5	10,2	859	Central-East community
Almería	5	11,6	1026	South community
Burgos	5	13,3	109	North community
Cáceres	5	12,1	1212	Central-West community
Cádiz	5	9,4	48	South community
Castellón/Castelló	5	12,3	267	Northeast community
Cuenca	5	13,3	3	Central-East community
Murcia	5	10,4	995	South community
Cantabria	5	12,6	422	Central-West community
Segovia	5	13,3	44	Central-West community
Tarragona	5	12,5	195	Northeast community
Badajoz	4	9,7	1209	South community
Ciudad Real	4	11,1	4	Central-East community
Coruña, A	4	7,3	57	Northwest community
Huesca	4	8,7	162	Northeast community
Jaén	4	9,6	11	South community
Lleida	4	8,9	118	Northeast community
Rioja, La	4	8,4	23	North community
Palencia	4	10	132	Central-West community
Pontevedra	4	7,3	57	Northwest community
Teruel	4	10,6	2	Northeast community
Balears, Illes	3	7,5	0	Central-East community
Huelva	3	7	906	South community
Palmas, Las	3	5,9	3	Canary Islands community
Soria	3	8,3	0	Central-East community
Girona	2	2,9	0	Northeast community
Guadalajara	2	3,6	0	Central-East community
Santa Cruz de Tenerife	2	2,7	0	Canary Islands community
Melilla	2	3,9	0	South community

Ceuta	1	1,2	0	South community

Community structure from the EDN: hierarchical clustering

When testing for communities and modularity in the EDN, as expressed in the main paper, a total of seven communities arises. Hierarchical clustering dendrogram (Fig S6) shows how provinces are grouped by a similarity degree (y axis), forming wider groups as the y axis ascends. Finally, each group of provinces conforms the branch and leaf of a tree. The colors used to label communities are the same than in the main paper, this is, from left to right: south, north-east, Canary Islands, north-west, north, central-east and central-west communities

From the dendrogram we can derive some conclusions: first of all, the south community is differentiated from the rest of the network. Only two provinces bridge the south community with the network, and it is the biggest community (12 members,) which makes this community one of the most independent and difficult to reach from any point of the network. It is no surprise that 7 provinces of this community score higher in betweenness, since travelling (in and out) is very costly in terms of number of steps.

The second main branch, encompassing the rest of the provinces, is first split up leaving the north-east community as an independent group. This region has some differential properties i.e., no clear mobility hub emerges (not even Barcelona, 2nd largest city in Spain), but a rather horizontal relation between all provinces emerges.

Finally, the las two branches, central east and west, even forming two groups, share a high degree of similarity and remain utterly proximal. It could barely conform an unique group. Indeed, if we look at figure 6 in the main text, these two communities have the lesser mean differences in CI14 curves between its members and the rest. Encompassing most of the peninsula and many different regions, it points towards a differentiated dynamic when compared with the rest.

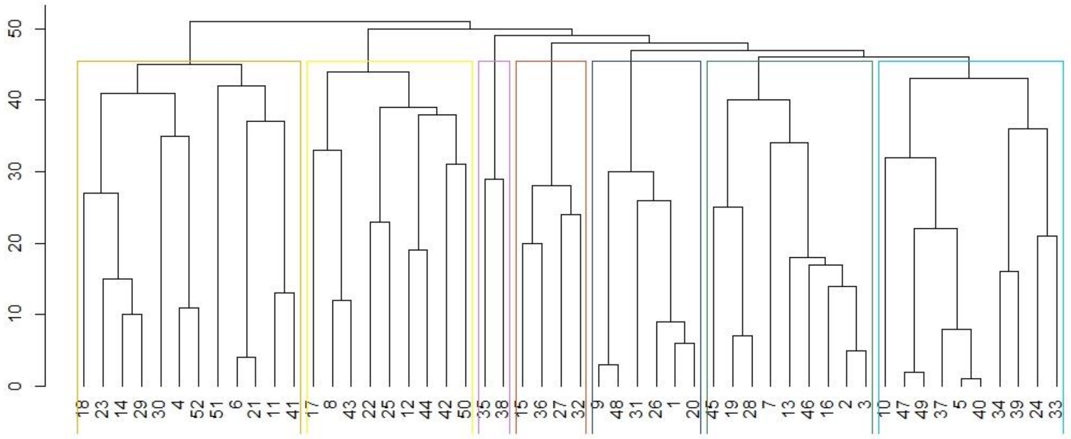

Figure S6. Hierarchical clustering dendrogram from the ED.
